# The NASOROSSO (Rednose) Project: An Italian Study on Alcohol Consumption in Recreational Places

**DOI:** 10.3390/ijerph10051665

**Published:** 2013-04-24

**Authors:** Roberta Pacifici, Andrea Pierantozzi, Rita Di Giovannandrea, Ilaria Palmi, Luisa Mastrobattista, Claudia Mortali, Simona Pichini

**Affiliations:** 1 Drug Abuse and Doping Unit, Department of Therapeutic Research and Medicines Evaluation Istituto Superiore di Sanità, Roma 00161, Italy; E-Mails: roberta.pacifici@iss.it (R.P.); rita.digiovannandrea@iss.it (R.D.G.); ilaria.palmi@iss.it (I.P.); luisa.mastrobattista@iss.it (L.M.); claudia.mortali@iss.it (C.M.); 2 Department of Laboratory Medicine, Tor Vergata University, Roma 00133, Italy; E-Mail: nonsolonumeri@gmail.com

**Keywords:** Breath Alcohol Consumption (BAC), youngsters, drivers, nightclubs, Italy

## Abstract

The Nasorosso project of the Italian Youth Department and the National Institute of Health, aimed to raise awareness about drinking and driving under the influence of alcohol among club goers with a series of initiatives. Within the framework of the project, blood alcohol concentration (BAC) was measured on 106,406 individuals before and after clubbing in 66 different recreational sites from 11 Italian provinces, over 16 months. Participating individuals were interviewed regarding sociodemographic and environmental characteristics and alcohol intoxicated people were offered to be taken home. The BAC median at the club entry was 0.26 g/L with 65.3% subjects showing a BAC value under the driving legal limit of 0.5g/L. At the exit from clubs, BAC median value rose to 0.44 g/L and subjects with BAC value under the legal limit decreased to 54.9%. Being male, aged between 18 and 34 years with a diploma, being a drinker and entering the disco with a BAC already beyond the legal limit predicted a BAC value beyond 0.5 g/L at exit from the recreational place. Conversely, being a driver, being a student and exiting from the disco before 4 a.m. reduced the probability of having a BAC higher than 0.5 g/L at the end of the night. Health policies to prevent harmful use of alcohol in young people should continue to offer targeted information/ prevention; in order to steadily increase the awareness of the dangers and the damages of excessive use of alcohol.

## 1. Introduction

The WHO European Region is the area with the highest consumption of alcohol in the World, with a prevalence of episodic intoxication in more than 20% of adults. After a slight decrease at the beginning of the 1990s, alcohol use in the European Region increased again in the 21st century to around an annual level of 9.5 L/capita. Whereas consumption varies greatly among countries, in 2011 the European average of pure alcohol annual consumption per person 14 years old or older has been 9.2 litres [1,2,3,4].

With respect to youngsters, the European School Survey Project on Alcohol and Other Drugs estimated that in 2011 an average of 87% of 15–16 year old European youngsters drank alcohol at least once during their lifetime with 57% having drank in the last month and 39% reporting binge drinking in the last 30 days [5]. Concerning European young adults, the highest alcohol consumption is generally reported for the age 25–39 year old range, which also shows the maximum number of episodes of binge drinking in the last months [6].

Over the past decade the amount of alcohol consumed decreased significantly in Italy starting from about 8 litres *per capita* per year at the beginning of new century to reach the value of 6.1 litres *per capita* per year in 2011 [7].

The Italian Institute of Statistics (ISTAT) estimated that, in 2011, 66.9% of the Italian population over the age of 14 consumed alcohol at least once a year with a growing proportion of people drinking not only at mealtimes. Episodes of binge drinking are still limited during first adolescence: 1.5% for 11–15 years old, but they increase to a 7.4% and 14.8% respectively at 16–17 and 18–19 years old, reaching a maximum of 15.1% in young adults of 18–24 years of age and decreasing to a 11.2% at 25–44 years of age [8].

Similar to the European Region situation, a significant gender difference related to harmful use of alcohol has been reported in the Italian peninsula. The 2012 ISTAT data revealed that 23.7% males present risky drinking behaviors *vs.* 7.0% females and this significant difference is evident in adolescence (14.1 *vs.* 8.4% in 11–17 years of age) young adulthood (22.8 *vs.* 8.4% in 18–25 years of age) and adulthood (20.5 *vs.* 5.9% in 25–44 years of age) [8].

Furthermore, the most recent national data on alcohol consumption shows that certain risk-taking behaviours are more common among those who more frequently attend nightclubs and recreational sites, affecting mainly males in every age group. Indeed, it has been observed that among the 18–24 years old people who go clubbing, 41.3% males and 16.8% females binge drank *vs.* the 9.5% males and 4.3% females who do not frequent the clubs [8]. Similar figures were reported for adolescents, young adults and adults up to 44 years of age who frequently participated in concerts and sport events [8].

The harmful consumption of alcohol in recreational situations such as the above mentioned ones has been associated with road traffic crashes [9,10,11,12,13,14].

In fact, 30–40% of drivers’ deaths in the European Union were caused by driving under the influence of alcohol [15]. In addition, the majority of alcohol-related accidents seem to occur at night and during weekends and the accidents have been associated to alcohol consumption as well as tiredness and a prolonged time spent at discos [16,17,18,19].

In this context, young drivers are the age group with the highest car accident risks, not only due to their limited driving experience, but also for driving or sitting in the passenger seat after alcohol consumption at recreational sites [20,21,22,23,24].

In the Italian peninsula, a recent study estimated that consumption of any quantity of alcohol within six hours prior to driving was associated with 2.25 fold increase in the risk of road traffic crashes [25]. In accordance with this, Trerotoli and colleagues showed that in 23.5% of 217 young Italian subjects going clubbing at night, blood alcohol concentration (BAC) was higher than 0.5 g/L (the maximum allowed BAC value for driving as legislated in a number of countries including Italy for adult drivers) when they left the disco [26].

At the end of 2008, the Italian Youth Department, seriously concerned with the problem of drinking and driving in club goers, in cooperation with the National Institute of Health (Istituto Superiore di Sanità, ISS), funded the Nasorosso (red nose) national project. The aim of the project was to raise awareness among youngsters, young adults and adults who go clubbing on alcohol use in recreational situations and driving under the influence of alcohol. All these activities were carried out through disseminating information, distributing information materials, supporting and counselling individuals attending recreational sites and people working at the clubs and clubs’ bars, creating networks of information and a specific website [27]. The project also involved trained staff who tried to discourage individuals who went clubbing from drinking and driving afterwards by offering to the alcohol intoxicated people, who declared to drive a car that night to be taken back home. To do this, club goers who gave consent and agreed to be interviewed, had their BAC measured when they arrived at the disco and when they left.

In this paper we present the sociodemographic characteristics of interviewed individuals in addition to their BAC values as an objective marker to disclose independent factors associated to harmful use of alcohol in recreational situations. Our aim was also to focus on the main characteristics of people with BAC values exceeding legal limits for driving, as possible target for preventive public health initiatives and strategies to avoid harmful use of alcohol.

## 2. Methods

### 2.1. Premises, Sampling and Subjects

The Nasorosso project was a multidisciplinary, multicentric, cross-sectional study carried out in 11 Italian provinces: Milano (Lombardy), Torino (Piedmont), Padova (Veneto) and Trieste (Friuli-Venezia-Giulia) in the northern part of the peninsula; Roma (Lazio), Viterbo (Lazio), Frosinone (Lazio) and Pescara (Abruzzo) in the centre; Napoli (Campania), Foggia (Puglia) and Cosenza (Calabria) in the south of the country. Subsequently, in each of the eleven selected provinces an Operating Unit of 3–5 people was organized which was responsible for the local project coordination.

Contacting the chambers of commerce, police forces and provincial youth political and recreational associations, nightclubs and recreational sites (e.g., pubs with music) were listed for each province. At the end of sampling, 66 different premises were identified: four in Milano and Torino, seven in Padova, five in Trieste, seven in Roma and Frosinone, five in Viterbo, three in Pescara, five in Napoli, 11 in Foggia and eight in Cosenza.

With respect to representativeness of the chosen provinces, even if the Nasorosso was an exploratory cross-sectional project, we set a minimum threshold of sample size considering about 40 million as the number of people who could potentially fall within the detection plane. Thus setting an estimation error sample to 0.5%, with a prevalence of subjects with alcohol over the value 0.5 g/L to 30%, we could estimate a minimum number of studied subjects in 32,268 individuals.

Following these essential characteristics, the three most important Italian cities (Milano, Roma and Napoli) representing the capitals of North, centre and South were selected together with other cities with popular premises open all year which agreed in joining the project. The proportion of subjects contacted by the survey compared to residents of the different provinces considered by the survey were between 0.41 to 1.61% in the big cities (Torino, Milano, Roma and Napoli) and between 1.57 to 6.51% in the medium and little towns for a global value of 1.34%.

It was decided to monitor 100 nights in total, with an average of 10 nights in each area from February 2010 to June 2011 and more than 100,000 subjects were expected to be contacted. At the start of the project, ISS organized an advertising campaign on a specific website [27], on city guides and local publications specifically dealing with the nightlife world.

At the entrance to the premises (discos or other recreational sites, e.g., pubs, night bars, *etc.*) the staff from each Operating Unit described the project to the entering people, counselling and providing a brief intervention on the harmful use of alcohol and road safety. The ones who wished to join the project were asked to sign an informed consent, to fill a structured questionnaire with the help of an interviewer, to undergo voluntary BAC test at the entry and at the exit of the premise and to be taken back home if they declared to drive a car that night and BAC value was beyond the legal limit for driving.

### 2.2. Questionnaire

The questionnaire had three sections, the first, self-filled, about (i) personal details: gender, place and date of birth, education level, social and occupational status, living conditions. In this respect, the following variables were defined: “occupational status” meaning that at the time of interview the person was employed (yes) or not (no); “educational level” meaning that at the time of interview the individual concluded the middle education (five years of primary school and three additional years of “middle” school) diploma (high school) or degree (university studies); “student” meaning that at the time of interview the individual was studying for any educational level (yes) or not (no); “marital status” meaning that at the time of interview the individual was married and living with the partner (yes) or was not married but single, separated or divorced living without any partner (no); “drinker” meaning that the individual was not abstinent and drinking or with the intention to drink at least one drink at time of interview (yes); “car driver” was an individual who came to the premise and came back from it driving a car eventually also bringing other people in the car (yes).

Second and third section, filled out by an interviewer, about (ii) drinking habits, BAC measurement at the entry and at the exit, (iii) coordinates of the respective premise (city, province, name of premise) and finally time of entry to the premise and time of exit from it.

### 2.3. Alcohol Test

The AlcoQuant Breathalyzer^®^ 6020 (Cozart Italia, Rome, Italy) was used to measure breath alcohol concentration (BrAC). It is a last-generation device using electrochemical sensors specifically developed to respond to alcohol test. These sensors can distinguish and eliminate factors that can invalidate the measured value (e.g., peppermint or eucalyptus fragrance or cigarette smoke). The measurement of BrAC involves the application of a disposable mouthpiece to maintain proper hygiene and assure a proper measurement of alcohol test. For legal purposes, the analyzer is set-up to convert the measured BrAC in a BAC value, the one appearing on the display of the analyzer. This latter value is legislated in Italy with the article No. 186 of the new street code (law No. 120 of the official Italian Gazette No. 175, 29 July 2010). For adult drivers the legal BAC limit to drive a car was established at 0.5 g/L, while for young drivers (within three years of driving license) and professional drivers it was set 0.0 g/L. Above those limits, the driver is deemed as “ethanol intoxicated” and “driving under the influence of ethanol”, subject to administrative or criminal sanctions (as a function of BAC value) and subsequent withdrawal of the driver license.

### 2.4. Statistical Analysis

An initial descriptive analysis was applied to the studied population. BAC was categorized according to the more recent Italian street code (Law 120/July 2010) using the cut-off of 0.5 g/L, above which a person is considered not able to drive. The normal distribution of continuous variables were evaluated with Kolmogorov-Smirnov test. Parametric (independent *t*-test) and non-parametric (Mann-Whitney test) tests were used to evaluate the differences between groups. Percentages were evaluated with binomial exact test. Logistic regressions were performed to assess the contribution, in terms of odds ratio, of each variable in predicting a BAC value beyond the legal limit. A *p*-value < 0.05 was considered statistically significant. All the analyses were carried out using the open source software R [28].

## 3. Results

### 3.1. Sociodemographic and Environmental Characteristics of Study Population

A total of 106,406 subjects were recruited and interviewed within the framework of the study. Table 1 shows the sociodemographic and environmental characteristics of all the participants, according to the city where they were enrolled. Significant statistical differences were observed among the different areas of data collection with respect to the mean values of all the participants from all the cities.

**Table 1:** Sociodemographic and environmental characteristics of all the participants, according to the city where they were enrolled.

Variables	Sex ^a^		Age ^b^	Occupational status ^a^	Educational level ^a^			Student ^a^	Marital status ^a^	Drinker ^a^	Car driver ^a^
	Male (%)	Female (%)	Mean ± SD	Yes (%)	Middle^§^ (%)	Diploma (%)	Degree (%)	Yes (%)	Yes (%)	Yes (%)	Yes (%)
**All the cities** **(N = 106,406)**	**70.1**	**29.9**	**26.3 ± 6.2**	**65.7**	**12.3**	**64.3**	**23.4**	**39.6**	**4.4**	**95.1**	**52.7**
**Cosenza** **(N = 5,759)**	69.2 ^2,5,6,7,9–11^	30.8 ^2,5–7,9–11^	24.6 ± 5.8 ^2,3,5–7,8–11^	50.5 ^2–11^	11.3 ^2,3,5–11^	78.9 ^2–11^	9.8 ^2–11^	47.7 ^2,3,6–11^	1.6 ^2–11^	98.8 ^2–10^	86.7 ^2–11^
**Foggia** **(N = 7,983)**	65.7 ^1,3–10^	34.3 ^1,3–10^	26.4 ± 6.7 ^1,3–7,10,11^	60.0 ^1,3–11^	20.0 ^1,3–11^	60.5 ^1,3–5,7–11^	19.5 ^1,3–8,10^	36.2 ^1,4–11^	9.5 ^1,3–11^	96.2 ^1,3–9,11^	49.6 ^1,3–6,8,10^
**Frosinone** **(N = 9,440)**	68.7 ^2,5–7,9–11^	31.3 ^2,5–7,9–11^	27.5 ± 6.7 ^1,2,4–6,8–11^	63.9 ^1,2,4–9^	10.2 ^1,2,4–11^	64.2 ^1,2,6–11^	25.6 ^1,2,4–9,11^	36.6 ^1,4–11^	8.2 ^1,2,4–11^	80.1 ^1,2,4–11^	70.7 ^1,2,4–11^
**Milano** **(N = 8,486)**	69.6 ^2,5–7,9–11^	30.4 ^2,5–7,9–11^	24.4 ± 5.5 ^2,3,5–11^	55.6 ^1–3,6–11^	12.3 ^2,3,5,7–11^	63.8 ^1,2,5–7,9–11^	23.9 ^1–3,5,6,8–11^	48.5 ^2,3,6–9,11^	3.5 ^1–3,5,7,11^	97.5 ^1–3,6–8,10,11^	47.8 ^1–3,5,6,9,10^
**Napoli** **(N = 8,519)**	62.9 ^1–4,6–11^	37.1 ^1–4,6–11^	26.0 ± 5.0 ^1–4,6,7^	54.6 ^1–3,6–11^	4.4 ^1–4,6–11^	64.9 ^1,2,4,6–11^	30.7 ^1–4,6–11^	47.1 ^2,3,6–11^	2.4 ^1–4,6,7,9,10^	96.9 ^1–3,6–8,10,11^	46.0 ^1–4,6–9,11^
**Padova** **(N = 14,122)**	67.4 ^1–5,7–11^	32.6 ^1–5,7–11^	27.0 ± 7.0 ^1–5,7–1^	68.4 ^1–5,8–11^	12.9 ^1–3,5,7–11^	59.5 ^1,3–5,7,8,9,11^	27.6 ^1–5,7,9–11^	39.8 ^1–5,8–10^	4.2 ^1–3,5,7,8,10,11^	95.8 ^1,3–5,7–9,11^	51.8 ^1–5,7,8–11^
**Pescara** **(N = 10,983)**	71.6 ^1–6,8,9,11^	28.4 ^1–6,8,9,11^	27.6 ± 6.5 ^1,2,4–6,8–11^	70.3 ^1–4,5,9–11^	8.8 ^1–6,9,10^	67.3 ^1–6,8–11^	23.9 ^1–3,5,6,8–11^	38.8 ^1–5,9,10^	5.4 ^1–6,8–11^	94.7 ^1–6,8–11^	48.2 ^1,3,5,6,9,10^
**Roma** **(N = 8,221)**	59.8 ^2,5,6,7,9–11^	40.2 ^2,5,6,7,9–11^	26.2 ± 5.7 ^1,3,4,6,7,10,11^	72.7 ^1–6,9–11^	9.0 ^1–6,9,10^	62.9 ^1–3,5–7,10,11^	28.1 ^1–5,7,9–11^	38.2 ^1–6,9,10^	3.1 ^1–3,6,7,9^	93.2 ^1–7,9–11^	48.0 ^1–6,9,10^
**Torino** **(N = 17,125)**	80.6 ^1–8,10,11^	19.4 ^1–8,10,11^	26.2 ± 6.0 ^1,3,4,6,7,10,11^	76.3 ^1–8,10,11^	17.2 ^1–8,10,11^	62.6 ^1–7,10,11^	20.2 ^1,3,4–8,10^	31.1 ^1–8,10,11^	4.0 ^1–3,5,7,8,11^	97.4 ^1–3,6–8,10,11^	50.0 ^1,3–8,10^
**Trieste** **(N = 5,857)**	71.7 ^1–6,8,9,11^	28.3 ^1–6,8,9,11^	25.7 ± 6.0 ^1–4,6–9^	63.0 ^1,2,4–9^	15.6 ^1–9,11^	58.8^1–5,7–9,11^	25.6^1,2,4–9,11^	49.3^1–3,5–9,11^	3.4^1–3,5–7,11^	95.6^1,3–9,11^	45.0^1–4,6–9,11^
**Viterbo** **(N = 9,911)**	65.9 ^1,3–10^	34.1 ^1,3–10^	25.7 ± 5.4 ^1–4,6–9^	65.9 ^1,2,4–9^	9.1 ^1–6,10,11^	70.4^1–10^	20.5^1,3–8,10^	38.8^1–5,9,10^	2.4^1–4,6,7,9,11^	98.5^2–10^	49.0^1,3,5,6,10^

^a^ binomial exact test, ^b^ independent *t*-test. statistically different (*p* < 0.05) from: ^1^ Cosenza, ^2^ Foggia, ^3^ Frosinone, ^4^ Milano, ^5^ Napoli, ^6^ Padova, ^7^ Pescara, ^8^ Roma, ^9^ Torino, ^10^ Trieste, ^11^ Viterbo. ^§^ Middle education includes five years of primary school and three additional years of “middle” school.

70.1% of the interviewed subjects were males with the lowest percentage in Viterbo (65.9%) and Rome (59.8%) and the highest in Turin (80.1%). The mean age of the individuals was 26.3 years, with youngest people in Milan (mean 24.4 years) and the oldest in Pescara (mean 27.6 years). On the average, 65.7% of subjects were employed, starting from 50.5% in Cosenza, in the southern part of Italy to 76.3% in Turin, in northern Italy. More than 60% of the people had a secondary school diploma and only 23.4% were university graduates, with minimal values in two cities, Cosenza (9.8%) and Foggia (19.5%), in the very south of the peninsula but with a maximum of 30.7% in Naples, the biggest city of Southern Italy. 39.6% of respondents were students. More than 90% of those who were interviewed were not married. This value was quite homogeneous in all the cities where the survey took place. 95.1% of the recruited subjects declared themselves to be alcohol drinkers. Among those who were interviewed, half (52.7%) were car drivers. The highest percentages of drivers were recorded in Cosenza (86.7%) and Frosinone (70.7%), the lowest ones in Naples (46%) and Trieste (45%).

### 3.2. Measurement of Blood Alcohol Concentration in the Study Population

Table 2 reports all the information concerning the measurement of BAC in the recruited participants from the different cities.

BAC was mainly detected between midnight and 2 a.m. (46.6%), because this time interval was the one with the largest gathering of people at recreational premises while BAC measurement at the exit from discos and other recreational sites was mainly performed between 2 and 4 a.m. (46.4%). On average, 70% of the interviewed subjects had one BAC measurement while 30% underwent the BAC test both, at entry to and the exit from the premises. The highest percentage of double measurements was recorded in Foggia (74.7%) and in Padova (73.9%), the lowest in Frosinone (3.9%) and in Viterbo (6.4%).

The BAC median at the club entry was 0.26 g/L (0.32 g/L for males and 0.13 g/L for females) with a mean values at 0.49 g/L (0.54 g/L for males and 0.37 g/L for females) with a maximum median values of 0.61, 0.60 and 0.50 g/L in Viterbo, Torino and Milano (confirmed by the highest mean values) and a minimum median of 0.0 g/L in Napoli. More interestingly at the club entry, an average of 65.3% subjects showed a BAC value under the legal limit with a maximum of 85.7% in Napoli and a minimum of 44.1% in Viterbo.

At the exit from clubs, BAC median rose to 0.44 g/L (0.48 g/L for males and 0.31 g/L for females) with mean value at 0.61 g/L (0.65 g/L for males and 0.51 g/L for females) confirming Viterbo, Torino and Milano as the cites with maximum median values: 0.65, 0.59 and 0.72 g/L, respectively. Subjects with BAC value under the legal limit decreased to 54.9%, confirming a minimum percentage of 40.8% in Viterbo and a maximum of 74.9% in Padova.

**Figure 1:**
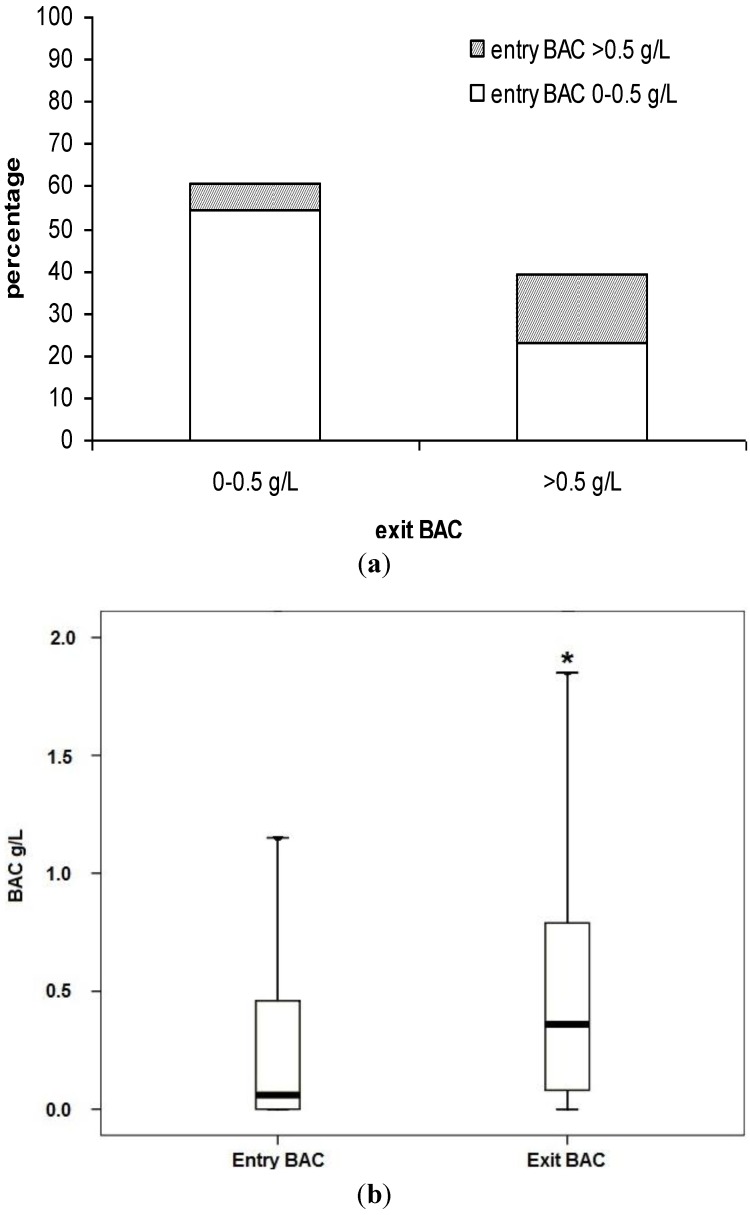
Comparisons of BAC values measured at the same people at the entry to the clubs and at the exit from them and box plot of BAC value at entry to the clubs and at exit from them (delta value: 0.24 g/L; * *p* < 0.0001).

On 32.020 % of individuals BAC measurement could be performed at the moment of entering the club and at the time of exit. Figure 1 shows the obtained results. At the exit, 60.6% of above-reported subjects presented a BAC value within the legal limits. Inside this group, a 89.5% also had showed a BAC within 0.5 g/L at the entry to the club. 

**Table 2:** Blood Alcohol Concentration (BAC) in all the participants, according to the city where they were enrolled.

Variables	Time of BAC detection at the entry ^a^			Time of BAC detection at the exit ^a^			Number of detection ^a^	
	<0.0 a.m. (%)	0.0–2.0 a.m. (%)	>2.0 a.m. (%)	<2.0 a.m. (%)	2.0–4.0 a.m. (%)	>4.0 a.m. (%)	One (% entry or exit)	Two (% entry and exit)
**All the cities ** **(N = 106,406)**	28.3	46.6	25.1	34.5	46.4	19.1	69.9	30.1
**Cosenza ** **(N = 5,759)**	14.1 ^2–7,9–11^	58.1 ^2–11^	27.8 ^2–11^	21.8 ^2–11^	64.3 ^2–11^	13.9 ^2–11^	79.2 ^2–11^	20.8 ^2–11^
**Foggia ** **(N = 7,983)**	33.4 ^1,3–,11^	42.8 ^1,3–11^	23.8 ^1,3–11^	39.3 ^1,3–11^	32.3 ^1,3–11^	28.4 ^1,3–9,11^	25.3 ^1,3–5,7–11^	74.7 ^1,3–5,7–11^
**Frosinone ** **(N = 9,440)**	48.0 ^1,2,4–11^	49.6 ^1,2,4–11^	2.4 ^1,2,4,5,7–11^	96.6 ^1,2,4–11^	2.9 ^1,2,4–11^	0.5 ^1,2,4,5,7–11^	96.1 ^1,2,4–11^	3.9 ^1,2,4–11^
**Milano ** **(N = 8,486)**	30.7 ^1–3,5–11^	38.9 ^1–3,5–11^	30.4 ^1–3,5–11^	43.7 ^1–3,5–11^	44.9 ^1–3,5–11^	11.4 ^1–3,6–11^	76.6 ^1–3,5–11^	23.4 ^1–3,5–11^
**Napoli ** **(N = 8,519)**	4.1 ^1–4,6–11^	79.7 ^1–4,6–11^	16.2 ^1–4,6–11^	11.4 ^1–4,6–11^	78.1 ^1–4,6–11^	10.5 ^1–3,6–11^	58.6 ^1–4,6–11^	41.4 ^1–4,6–11^
**Padova ** **(N = 14,122)**	66.3 ^1–5,7–11^	32.2 ^1–5,7–11^	1.5 ^1,2,4,5,7–11^	88.3 ^1–5,7–11^	11.4 ^1–5,7–11^	0.3 ^1,2,4,5,7–11^	26.1 ^1,3–5,7–11^	73.9 ^1,3–11^
**Pescara ** **(N = 10,983)**	0.9 ^1–6,8,10,11^	63.9 ^1–6,8–11^	35.2 ^1–6,8–11^	4.7 ^1–6,8–11^	72.4 ^1–6,8–11^	22.9 ^1–6,8–11^	88.5 ^1–6,8–11^	11.5 ^1–6,8–11^
**Roma ** **(N = 8,221)**	14.4 ^2–6,7,9–11^	65.4 ^1–7,9–11^	20.2 ^1–7,9–11^	17.4 ^1–7,9–11^	62.5 ^1–7,10,11^	20.1 ^1–7,9,10^	60.2 ^1–7,9–11^	39.8 ^1–7,9–11^
**Torino ** **(N = 17,125)**	0.1 ^1–6,8,10,11^	48.6 ^1,2,4–8,10,11^	51.3 ^1–8,10,11^	2.3 ^1–8,10,11^	61.7 ^1–7,10,11^	36.0 ^1–8,10,11^	85.2 ^1–8,11^	14.8 ^1–8,11^
**Trieste ** **(N = 5,857)**	7.5 ^1–9,11^	33.8 ^1–9,11^	58.7 ^1–9,11^	19.1 ^1–9,11^	52.1 ^1–9,11^	28.8 ^1,3–9,11^	85.6^1–8,11^	14.4 ^1–8,11^
**Viterbo ** **(N = 9,911)**	2.4 ^1–10^	51.6 ^1–10^	46.0 ^1–10^	26.3 ^1–10^	54.8 ^1–10^	18.9 ^1–7,9,10^	93.6^1–10^	6.4 ^1–10^
**Variables**	**BAC value at the entry**				**BAC value at the exit**			
	**Median ^b^**	**Mean ± SE ^c^**	**0–0.5 (%) ^a^**	**>0.5 (%) ^a^**	**Median ^b^**	**Mean ± SE ^c^**	**0–0.5 (%) ^a^**	**>0.5 (%) ^a^**
**All the cities** **(N = 106,406)**	**0.26**	**0.49± 0.002**	**65.3**	**34.7**	**0.44**	**0.61± 0.002**	**54.9**	**45.1**
**Cosenza** **(N = 5,759)**	0.41 ^2–9,11^	0.74 ± 0.017 ^2,3,5–8,10^	53.8 ^2–11^	46.2 ^2–11^	0.55 ^2–8,10,11^	0.82 ± 0.013 ^2–11^	47.8 ^2–11^	52.2 ^2–11^
**Foggia** **(N = 7,983)**	0.10 ^1,3–7,9–11^	0.37 ± 0.007 ^1,3–11^	75.2 ^1,3–7,9–11^	24.8 ^1,3,4–7,9–11^	0.33 ^1,4–7,9–11^	0.67 ± 0.012 ^1,3–8,10,11^	60.2 ^1,3–11^	39.8 ^1,3–11^
**Frosinone** **(N = 9,440)**	0.27 ^1,2,4–11^	0.60 ± 0.011 ^1,2,4–11^	64.5 ^1,2,4–11^	35.5 ^1,2,4–11^	0.30 ^1,4–11^	0.58 ± 0.012 ^1,2,4–9,11^	64.8 ^1,2,4–11^	35.2 ^1,2,4–11^
**Milano** **(N = 8,486)**	0.50 ^1,2,5–9,11^	0.77 ± 0.010 ^2,3,5–11^	50.0 ^1–3,5–11^	50.0 ^1–3,5–11^	0.72 ^1–3,5–11^	0.98 ± 0.018 ^1–3,5–11^	37.2 ^1–3,5–11^	62.8 ^1–3,5–11^
**Napoli** **(N = 8,519)**	0.00 ^1–4,7–11^	0.20 ± 0.006 ^1–4,6–11^	85.7 ^1–4,6–11^	14.3 ^1–4,6–11^	0.41 ^1–4,6–11^	0.61 ± 0.008 ^1–4,6,8–11^	56.7 ^1–4,6–11^	43.3 ^1–4,6–11^
**Padova** **(N = 14,122)**	0.04 ^1–4,7–11^	0.25 ± 0.003 ^1–5,7–11^	82.2 ^1–5,7–11^	17.8 ^1–5,7–11^	0.22 ^1–5,7–11^	0.36 ± 0.004 ^1–5,7–11^	74.9 ^1–5,7–11^	25.1 ^1–5,7–11^
**Pescara** **(N = 10,983)**	0.39 ^2–6,8,9,11^	0.61 ± 0.010 ^1,2,4–6,8–11^	57.1 ^1–6,8,9,11^	42.9 ^1–6,8,9,11^	0.46 ^1–6,8,1^°^,11^	0.59 ± 0.006 ^1–4,6,8,9,11^	53.7 ^1–6,8,9,11^	46.3 ^1–6,8,9,11^
**Roma** **(N = 8,221)**	0.11 ^1,3–7,9–11^	0.32 ± 0.005 ^1–7,9–11^	75.3 ^1,3–7,9–11^	24.7 ^1,3–7,9–11^	0.36 ^1,3–7,9–11^	0.47 ± 0.008 ^1–7,9–11^	62.1 ^1–7,9–11^	37.9 ^1–7,9–11^
**Torino** **(N = 17,125)**	0.60 ^1–8,10^	0.73 ± 0.007 ^2–8,10^	44.4 ^1–8,10^	55.6 ^1–8,10^	0.59 ^2–8,10,11^	0.67 ± 0.004 ^1,3–8,10,11^	42.8 ^1–8,10,11^	57.2 ^1–8,10,11^
**Trieste** **(N = 5,857)**	0.44 ^2,3,5,6,8,9,11^	0.53 ± 0.006 ^1–9,11^	56.8 ^1–6,8,9,11^	43.2 ^1–6,8,9,11^	0.46 ^1–9,11^	0.54 ± 0.011 ^1,2,4–6,8,9,11^	54.6 ^1–6,8,9,11^	45.4 ^1–6,8,9,11^
**Viterbo** **(N = 9,911)**	0.61 ^1–8,10^	0.70 ± 0.010 ^2–8,10^	44.1 ^1–8,10^	55.9 ^1–8,10^	0.65 ^1–10^	0.75 ± 0.007 ^1–10^	40.8 ^1–10^	59.2 ^1–10^

^a^ binomial exact test, ^b^ mann-whitney test, ^c^ independent *t*-test. statistically different (*p* < 0.05) from: ^1^ Cosenza, ^2^ Foggia, ^3^ Frosinone, ^4^ Milano, ^5^ Napoli, ^6^ Padova, ^7^ Pescara, ^8^ Roma, ^9^ Torino, ^10^ Trieste, ^11^ Viterbo.

The remaining 39.4% subjects had an exit BAC value beyond the legal limit. Of the latter, a 41.6% already has the BAC value beyond the legal limit at the club entrance. If only the car drivers were considered, their median BAC value at the entry to the premise was 0.20 g/L (mean at 0.44 g/L) and 0.35 g/L (mean 0.53 g/L) at the exit.

In Table 3 sociodemographic and environmental characteristics of all the participants are reported as a function of BAC values above the legal limit at the entry to the disco and at the exit from it, to examine which characteristics correlated best with high BAC, above the driving limit. Percentages were evaluated with binomial exact test. The entry level of BAC > 0.5g/L was considered as independent dichotomous variable.

**Table 3:** Sociodemographic and environmental characteristics of all the participants influencing BAC values measured at the entry to and at the exit from the discos (or other recreational sites).

Variable		ENTRY BAC > 0.5g/L	EXIT BAC > 0.5g/L	*p* value
Sex ^a^	M (%)	38.3	48.4	<0.001
	F (%)	26.0	36.2	<0.001
Age ^b^	<18	29.3	40.7	<0.001
	18–24	35.3	45.2	<0.001
	25–34	35.4	46.3	<0.001
	35–44	31.8	40.7	<0.001
	>45	27.2	33.3	<0.001
Occupational status ^a^	Yes (%)	36.0	45.5	<0.001
Educational level ^a^	Middle (%)	35.7	45.1	<0.001
	Diploma (%)	35.1	45.4	<0.001
	Degree (%)	31.1	42.4	<0.001
Entry time ^a^	<0.0 a.m. (%)	20.0	26.3	<0.001
	0.0–2.0 a.m. (%)	33.7	42.6	<0.001
	>2.0 a.m. (%)	55.1	57.0	0.102
Exit time ^a^	<2.0 a.m. (%)	14.8	31.3	<0.001
	2.0–4.0a.m. (%)	28.6	50.0	<0.001
	>4.0 a.m. (%)	33.4	56.8	<0.001
Student ^a^	Yes (%)	35.2	44.6	<0.001
Marital status ^a^	Yes (%)	31.1	42.1	<0.001
Drinker ^a^	Yes (%)	37.8	46.9	-
Car driver ^a^	Yes (%)	30.1	38.1	<0.001

^a^ binomial exact test, ^b^ independent *t*-test.

The most interesting observations on the Table data are the following:

1. Both at entry to and the exit from the disco a higher % of males showed BAC > 0.5g/L and for both genders a significantly higher percentage of “ethanol intoxicated” people was recorded at the exit from the disco than at the entrance.
2. A significantly higher percentage of people from all age groups, educational levels and occupational status came out from the recreational sites with BAC > 0.5 g/L. This was also the case for car drivers.
3. Entering the disco after midnight increased the percentage of people with BAC > 0.5g/L both, at entrance and at exit compared to the ones who entered the disco before midnight, but this was not true when people arrived after 2 a.m.;
4. The later the exit time, the higher the % of people with BAC beyond the legal limit.

In one word, independent from all sociodemographic and environmental characteristics, a higher percentage people showed a BAC value beyond the legal limit after they spent some time in a recreational site. To identify independent risk factors for BAC values beyond the legal limit at the end of the night logistic regressions were performed and are presented in Table 4.

**Table 4:** Logistic regressions to assess the contribution, in terms of odds ratio, of each sociodemographic and environmental characteristics of BAC beyond the legal limit at the exit from the discos (or other recreational sites).

Variable	EXIT BAC > 0.5g/L	
	OR (c.i. 95%)	*p*-value
*Sex (Male)*	1.768 (1.685–1.854)	<0.001
Age *		
<18	1.250 (0.999–1.564)	0.051
18–24	1.327 (1.124–1.567)	0.001
25–34	1.434 (1.219–1.687)	<0.001
35–44	1.220 (1.029–1.447)	0.022
*Occupational status*	0.990 (0.934–1.050)	0.745
Educational level *		
Middle	1.083 (1.003–1.170)	0.043
Diploma	1.142 (1.084–1.204)	<0.001
Exit time ***		
<2.0 a.m.	0.375 (0.353–0.398)	<0.001
2.0–4.0 a.m.	0.785 (0.742–0.831)	<0.001
*Time (hours) spent inside the premise*	1.001 (0.999–1.002)	0.152
*Entry BAC > 0.5 g/L^§^*	5.586 (5.102–6.097)	<0.001
*Student*	0.894 (0.845–0.947)	<0.001
*Marital status*	1.122 (1.013–1.243)	0.027
*Drinker*	2.223 (1.745– 2.832)	<0.001
*Car driver*	0.515 (0.474–0.559)	<0.001

* reference category: >45 years. ** reference category: degree. *** reference category: exit time > 4.0 a.m. ^§^ calculated for people with double BAC measurement.

## 4. Discussion

Being male, aged between 18 and 34 years with a diploma, being a drinker and entering the disco with a BAC already beyond the legal limit predicted a BAC value beyond 0.5 g/L at exit from the recreational place. Conversely, being a driver, being a student and exit from the disco before 4 a.m. reduced the probability of having a BAC major than 0.5 g/L at the end of the night.

The Nasorosso project is the largest study ever carried out in Italy to raise awareness among youngsters, young adults and adults who go clubbing on alcohol use in recreational situations and driving under influence of consumed alcohol.

In addition to the large information campaign along the entire Italian peninsula for more than one year, the project gave the opportunity to interview more than 100,000 people going clubbing together with the objective assessment of their blood alcohol levels before and after the recreational event.

Statistical analysis of data highlighted a first important conclusion: discos or other nightlife facilities are no longer the only places where alcoholic beverages are consumed, since many people come into these places having already consumed alcohol.

The gender difference has an influence on the drinking habits of night clubs frequenters: Italian women tend to have a more responsible attitude and BAC values significantly lower than men on all occasions, whether or not they are drivers. This feminine trend was also confirmed by the fact that the provinces with a higher prevalence of male participants were the ones with higher BAC values.

Generally, the drivers (both men and women, young or not) showed a more responsible behaviour than non-drivers: their BAC tended to be lower than for non-drivers, though not always within the limits imposed by the law. In this context about one out of three drivers arrived at the disco with a BAC value greater than the one allowed by the latest street code, and about 15% had an “alarming” blood alcohol level (>0.9 g/L, data not shown). Leaving the nightclubs, 38% of surveyed drivers had a BAC higher than the 0.5 g/L and 18.5% had a BAC greater than 0.9 g/L.

Even the time of entry into disco influenced the BAC value of the respondents, regardless of the fact of being drivers or not. Those who entered the premises after 2.00 a.m., whether they were drivers or not, showed a BAC value higher than that of the people who entered before that time. These results indicate that club goers tend to drink outside nightclubs, or have the habit of moving from one premise to another consuming alcoholic beverages during the night. These results are in agreement with the study of Gallimberti *et al.* [29] on 845 Italian underage school students. Social, demographic and environmental factors that may raise the risk of Saturday night drinking and binge drinking were investigated. The independent variables that conferred a higher risk of drinking in underage students were older age groups, gender (males) and returning home after midnight. Spending time in bars or discos coincided with a two-fold or four-fold increase, respectively, in the risk of alcohol consumption.

Indeed, in our study, a higher percentage of people showed a BAC value beyond the legal limit at the exit from the disco, showing that the stay in the premise was another drinking occasion. This was true even in case of young adults- 18–24 years, for which street code rules are stricter: their BAC limit has to be 0.0 g/L, meaning that they should not drink at all if they drive.

Examining the data relating to road accidents in the provinces participating in the Nasorosso Project, it becomes clear that during 2010–2011 there was a substantial decrease in the number of car crashes and involved people (measured as injured and dead people) in all the provinces included in the project. Nonetheless, if considering other provinces from the same regions where project intervention was not performed, a decrease in car accidents was also observed with similar figures. In any case, it could be noted that some of the provinces included in the project (Turin, Trieste, Milan and Rome) were the ones with the highest significant decrease in mortality index in car crashes [30].

Although it is not possible to attribute the reduction of road accidents to the information initiatives/prevention-related activities of the project, it can be supposed that these initiatives helped to support the decreasing trend. On the other hand, it has to be said that this could be also attributed to the worldwide financial crisis period causing a reduced transport mobility.

Conversely, the ACI-ISTAT report also underlined that, notwithstanding the general significant decrease in percentage of accidents, the highest mortality index is still registered Friday, Saturday and Sunday night between 22 p.m. and 6 a.m. with Milano and Torino being the cities with the highest number of accidents and fatalities, together with the capital Rome [30].

In spite of these interesting results, confirmed by other studies and official reports, the present study has certain limitations that need to be addressed. First, participants’ recruitment was on a voluntary basis and it can be hypothesized that more intoxicated people, due to guilt feelings and lack of interest in the initiatives, refused to be interviewed and undergo BAC measurement. Second, due to the need to submit a brief questionnaire to people entering a recreational site for amusement, many other risk factors such as: use of licit and illicit drugs, tiredness at the entry to and the exit from the discos, which could have predicted (influenced) “ethanol intoxication” in a recreational night, could not be investigated.

Nonetheless, the huge sample size and its diffusion throughout the national territory support the study’s validity. The most important message for the Italian Youth Department which funded and commissioned this study is that the National Health System should focus its information campaigns and awareness initiatives on “risky drinking and driving” young adults and on adult educated men who go clubbing, especially in late hours [31].

## 5. Conclusions

In conclusion, it is important to remember that health policy towards youth should continue to offer targeted information/prevention, in order to steadily increase the awareness on the dangers and the damages of excessive o alcohol use, specifically during recreational events. It would be desirable, for example to propose and implement self-measurement of BAC not only in discos (as now legislated in the new street code of the Highway Code), but also in other recreational places. In addition, in future legislations the awareness and education of owners and employees of night life premises concerning drinking and driving should be emphasized.
